# High-Performance Polyimide Filaments and Composites Improved by O_2_ Plasma Treatment

**DOI:** 10.3390/polym10070695

**Published:** 2018-06-22

**Authors:** Fangbing Lin, Wei Li, Yusi Tang, Huiqi Shao, Chuanli Su, Jinhua Jiang, Nanliang Chen

**Affiliations:** 1 Engineering Research Center of Technical Textiles, Ministry of Education, Donghua University, Shanghai 201620, China; fangbinglin@tamu.edu (F.L.); weilichn@tamu.edu (W.L.); 1142002@mail.dhu.edu.cn (H.S.); 2160042@mail.dhu.edu.cn (C.S.); 2 College of Textiles, Donghua University, Shanghai 201620, China; 3 Department of Industrial and Systems Engineering, Texas A&M University, College Station, TX 77843, USA; 4 Shanghai YS Information Technology Co., Ltd., Shanghai 201100, China; 2140024@mail.dhu.edu.cn

**Keywords:** polyimide filaments, O_2_ plasma treatment, surface characteristics, adhesion, mechanical properties, composites

## Abstract

Interface issues urgently need to be addressed in high-performance fiber reinforced composites. In this study, different periods of O_2_ plasma treatment are proposed to modify twist-free polyimide (PI) filaments to improve hydrophilicity and mechanical and interfacial properties. Feeding O_2_ produces chemically active particles to modify the filament surface via chemical reactions and physical etching. According to the X-ray photoelectron spectroscopy (XPS) results, the PI filaments exhibit an 87.16% increase in O/C atomic ratio and a 135.71% increase in the C–O functional group after 180 s O_2_ plasma treatment. The atomic force microscope (AFM) results show that the root mean square roughness (Rq) of the treated PI filaments increases by 105.34%, from 38.41 to 78.87 nm. Owing to the increased surface oxygenic functional groups and roughness after O_2_ plasma treatment, the contact angle between treated PI filaments and water reduces drastically from the pristine state of 105.08° to 56.15°. The O_2_ plasma treated PI filaments also demonstrate better mechanical properties than the pristine PI filaments. Moreover, after O_2_ plasma treatment, the adhesion between PI filaments and poly(amic acid) (PAA) is enhanced, and the tensile strength of the polyimide/poly(amic acid) (PI/PAA) self-reinforced composites increases from 136 to 234 MPa, even causing the failure mode of the composite changes from adhesive failure to partly cohesive failure.

## 1. Introduction

Polyimide (PI) filaments as reinforcements for advanced composites have attracted considerable research attention for their favorable thermal stability, outstanding mechanical properties, excellent chemical and radiation resistance, and special dielectric properties [1,2,3,4,5]. For composites, good interfacial adhesion between fiber and matrix facilitates the load transfer efficiency and improves the strength and toughness of the composite materials [6,7,8,9]. However, the surface of the PI filaments is relatively smooth, chemically inert, and inherently hydrophobic, and thus, leads to very poor interfacial adhesion between PI filaments and the matrix, which limits the PI filaments as the reinforcement agent in advanced composites [10,11]. Therefore, optimizing the surface properties of PI filaments to improve the PI filaments/matrix adhesion is urgently needed to obtain high-performance composites.

Recently, to overcome the limitations of the poor adhesion of high-performance fibers, a variety of techniques have been used, such as the acid oxidation treatment, alkali solution treatment, gas-phase oxidation, heat treatment, polymerization treatment, cryogenic treatment, and plasma treatment [5,12,13,14,15,16,17,18,19]. Among these methods (see Table S1), plasma treatment only works on the uppermost layer of the material without compromising the bulk properties. This method is also time-saving, eco-friendly, effective and economical, and is especially suitable for continuous and large-scale processing of filaments and other textile structural materials [20,21,22]. To the best of our knowledge, the present reported plasma treatment of PI fiber mostly focused on the temperature-resistant properties of ordinary PI fiber or short PI fiber [23,24,25,26,27,28], and the mechanical properties of plasma treatment of high-performance PI filaments have never been reported. The high-performance PI filaments are highly promising for aerospace engineering, electric cables and other applications due to its continuity, ease of handling, and orientation selection during composites fabrication. Therefore, high-performance PI filaments, which are modified with good adhesion to the matrix, are in increasing demand for advanced composites.

In this study, O_2_ plasma treatments at various time intervals were applied to high-performance PI filaments (consisting of 20 single filaments) to enhance the adhesion property without sacrificing the mechanical properties. The effects of various O_2_ plasma treatments on morphologies, surface chemical compositions and wettability of PI filaments were investigated and theoretically analyzed. The obtained O_2_ plasma treated PI filaments showed excellent mechanical properties. Furthermore, the fabricated O_2_ plasma treated PI filaments/poly(amic acid) (PI/PAA) self-reinforced composites demonstrated better mechanical properties. These O_2_ plasma treated PI filaments are promising candidates for potential applications in advanced composites and aerospace applications.

## 2. Materials and Methods

### 2.1. Materials

The high-performance PI filaments, kindly provided by China Shino New Materials Company (Changzhou, China), were fabricated by 3,3′,4,4′-biphenyl tetracarboxylic dianhydride (BPDA) and *p*-Phenylenediamine (*p*-PDA) using a two-step spinning technology [29]. The diameter, density, tensile strength, modulus and breaking elongation of the individual PI filaments was 12.5 μm, 1.46 g/cm^3^, 3.0 GPa, 120 GPa, and 3.0%, respectively.

### 2.2. O_2_ Plasma Modified PI Filaments

**Figure 1:**
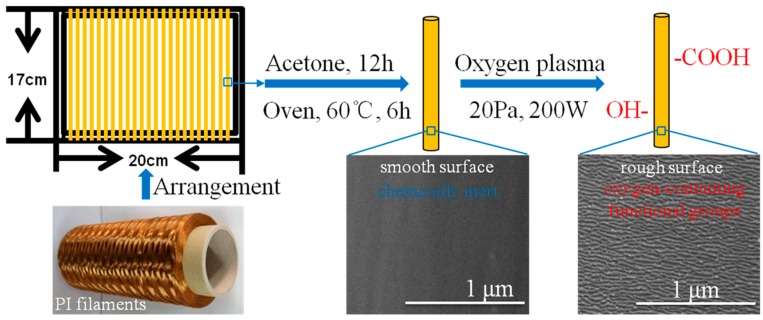
Specimen preparation and plasma treatment procedures of polyimide (PI) filaments.

The O_2_ plasma treatment procedures of PI filaments are described in Figure 1. First, to ensure all the samples were treated uniformly and thoroughly, the PI filaments were wound and fixed on a plastic frame with a dimension of 17 cm × 20 cm. Second, to eliminate the effect of contaminants, such as greases on the filament surface, the plastic frame with PI filaments were immersed into acetone solvent at 20 °C for 12 h, then cleaned by deionized water five times and dried in a vacuum oven at 60 °C for 6 h. Next, the plasma treatments were carried out in a plasma chamber of HD-300 equipment (ChangZhou ZhongKe ChangTai Plasma Technology, Changzhou, China) using O_2_ atmosphere. The treatment time was controlled at 60, 120, 180 and 240 s, under a fixed pressure of 20 Pa and a power of 200 W. After the treatments, the treated PI filaments were immediately sealed in a clean plastic bag for further experiments.

### 2.3. Preparation of PAA and PI/PAA Self-Reinforced Composite

The poly(amic acid) (PAA) was prepared by mixing equal molar ratios of dianhydride and diamines in dimethylacetamide (DMAc) solvent and stirring at 0 °C for 24 h to synthesize a BPDA/*p*-PDA solution. A yellow viscous PAA solution with 10 wt % solid content was obtained.

The pristine and O_2_ plasma treated PI filaments were fixed on a glass plate and dipped with 10 wt % PAA solution by a coating technology, followed by thermal imidization using the following process: (1) Heating to 80 °C (5 °C/min) and annealing for 1.5 h; (2) heating to 120 °C (5 °C/min) and annealing for 1 h; (3) heating to 160 °C (5 °C/min) and annealing for 1 h; (4) heating to 350 °C (5 °C/min) and annealing for 1 h; (5) cooling down to room temperature [30,31,32,33]. The manufactured composites had a thickness of ~100 um, and the fiber volume fraction in the composites was ~30%. 

### 2.4. Characterization

#### 2.4.1. Surface Morphology Observation by SEM

The surface morphologies of the pristine and O_2_ plasma treated PI filaments, as well as the tensile fracture of PI/PAA composites, were observed by field emission scanning electron microscope (FE-SEM, HITACHI S-4800, Tokyo, Japan) with an acceleration voltage of 5 kV. Specimens were coated with a thin layer of gold prior to the observation. Nanomeasurer software was used to measure the diameter of PI filament.

#### 2.4.2. Surface Topography Observation by AFM

The surface topography of PI filaments was conducted by atomic force microscope (AFM, Agilent 5500, Santa Clara, CA, USA) in air with a tapping mode. A silicon probe with a nominal resonance frequency of 190 kHz, and a force constant of 48 N/m (KS-Tap 190AL-G, KEYSIGHT Budget Sensors, Sofia, Bulgaria) was used in the dynamic AFM experiments. A single filament was fixed on a silicon chip with double-sided adhesive tape. The scanning scope was 4 μm × 4 μm and the surface roughness of PI filaments was analyzed using Pico Image Elements 7 software (Santa Clara, CA, USA).

#### 2.4.3. XPS Analysis

The surface chemical compositions of PI filaments were measured using X-ray photoelectron spectroscopy (XPS, ESCALAB 250, Thermo Electron VG Scientific, Waltham, MA, USA). The X-ray source was Al-Ka (1486.6 eV) and emitted photo-electrons were collected at a take-off angle of 45°. An energy step size of 0.05 eV and a pass energy of 20 eV were used to acquire the detailed spectra. The pressure within the XPS chamber was between 10^−7^ to 10^−8^ Pa, and the deconvolution analysis of C1s peaks was carried out using XPS-PEAK software (Waltham, MA, USA). The software had a Shirley background subtraction and a Gaussian-Lorentzian mix function. Each peak was fitted by Gaussian 70%–Lorentzian 30% mixture curves that were constrained in location and full width at half maximum (FWHM).

#### 2.4.4. Contact Angle Measurement

The contact angle of PI filaments was measured by the sessile drop method (OCA15 EC type tester, Beijing, China). Before the test, the PI filaments were arranged horizontally and closely parallel to each other to form a film-like plane [34]. Through an optical microscope equipped with a camera, the digital pictures of a drop of distilled water on the surface of individual filament were observed as the water droplets were kept on the surface of the samples for 60 s. The mean angle value on both sides of the distilled water droplets was adopted as the available contact angle.

#### 2.4.5. Mechanical Properties

The mechanical properties of PI filaments and PI/PAA composites were tested on an XQ-2 tensile tester (Shanghai Xusai Instrument Co., Shanghai, China) at a crosshead speed of 1 mm/min with a gauge length of 20 mm at 20 °C and 65% relative humidity. The tensile strength was obtained by loaded force/cross-sectional area of the sample, and the dimension of cross-sectional area was observed under a microscope (ECLIPSE LV100 POL, Nikon, Tokyo, Japan). At least 20 specimens were tested for each sample, and the means were calculated.

## 3. Results

### 3.1. Morphologies

**Figure 2:**
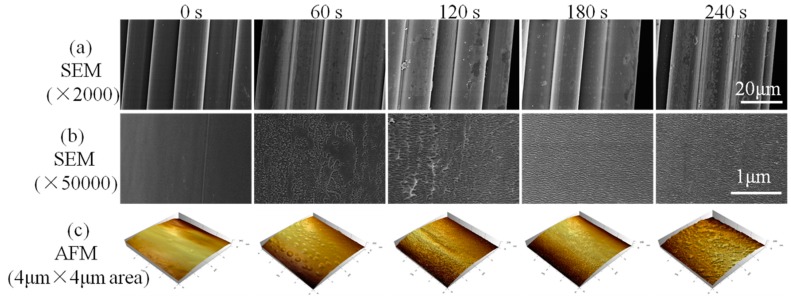
Surface morphologies of PI filaments with plasma treatment time of 0, 60, 120, 180 and 240 s, where (**a**) SEM images with 2000 magnification, (**b**) SEM images with 50,000 magnification and (**c**) 3D atomic force microscope (AFM) images with the scan size of 4 μm × 4 μm.

**Figure 3:**
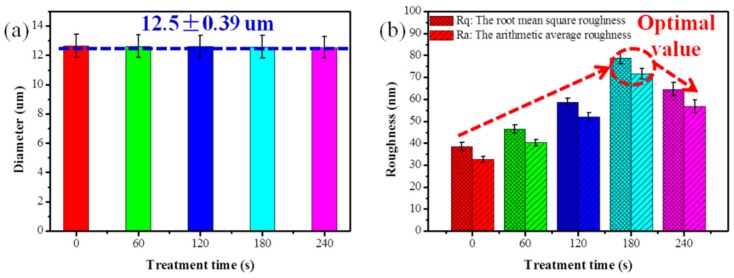
(**a**) Diameter of PI filament, (**b**) roughness of PI filament with plasma treatment time of 0, 60, 120, 180 and 240 s.

To study the effects of O_2_ plasma treatment on the high-performance PI filaments, a series of characterizations was carried out. One of the most important effects of O_2_ plasma induced on PI filaments is surface etching, which can roughen the filament surface. Figure 2 shows the surface of the pristine PI filaments is relatively clean and smooth. After being treated by O_2_ plasma for 60 s, a few visible but non-uniform etching spots are formed on the PI filament surface. More etching spots and protrusions can be observed on the surface of 120 s treated PI filament, while the etching spots are uneven and shallow in some areas. As the treatment time increases to 180 s, substantial, even-distributed and deep etching spots can be observed on the dramatically rough PI filament surface (see the SEM pictures in Figure S1, Supplementary Materials). However, carrying on plasma treatment to 240 s will destroy the filaments and lead to excessive etching spots on the surface (Figure 2a,b). After O_2_ plasma treatment, the diameter of PI individual filament did not change (Figure 3a).

AFM was applied to further examine the surface topographies of PI filaments with and without O_2_ plasma treatment. Corresponding to the SEM results, the surface of the pristine PI filament is relatively smooth, and the surface roughness of the O_2_ plasma treated PI filaments increases as the treatment time increases from 60 to 180 s, as shown in Figure 2c and Figure 3b. Moreover, the O_2_ plasma treated PI filaments for 180 s shows even-distributed and deep grooves, which lead to the root mean square roughness (Rq) increase up to 78.87 nm from 38.41 nm for the pristine PI filaments. However, as the O_2_ plasma treatment time increases to 240 s, the surface roughness decreases compared with the 180 s samples, which is ascribed to the excessive etching or ablation action on the PI filament surface. Thus, it can be concluded that a suitable O_2_ plasma treatment time of around 180 s is the optimal parameter in terms of the surface roughness. The rougher surface provides more capillary holes and facilitates the matrix molecules infiltration into the filaments, and thus, enhances the interfacial adhesion between the filaments and matrix.

### 3.2. Surface Chemical Composition Analysis

**Figure 4:**
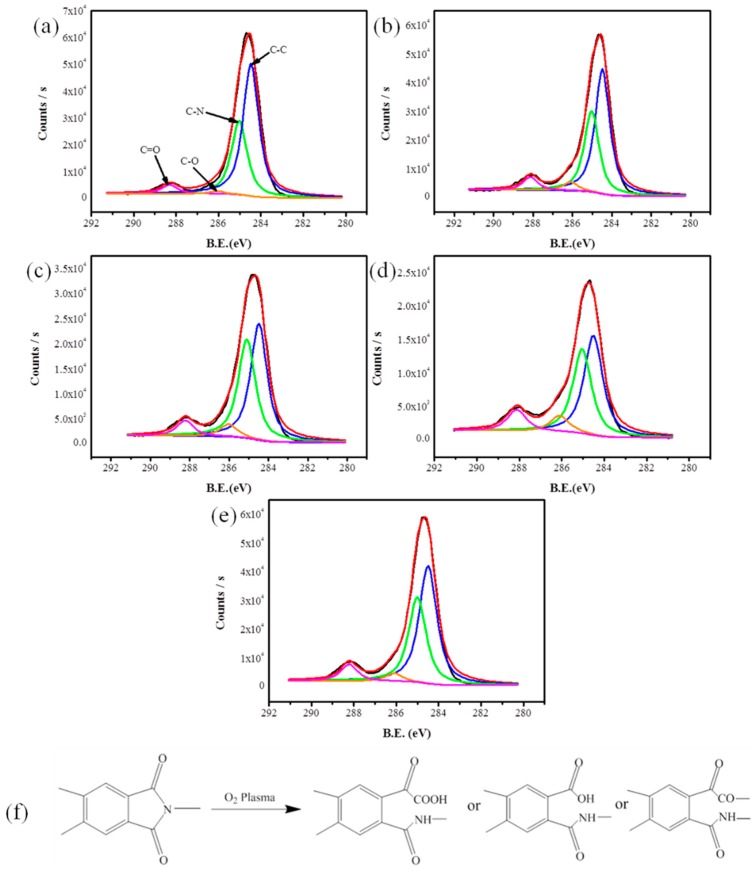
Deconvolution of the C1s peak of X-ray photoelectron spectroscopy (XPS) spectra of PI filaments: (**a**) pristine, O_2_ plasma treated with times of (**b**) 60 s, (**c**) 120 s, (**d**) 180 s and (**e**) 240 s, (**f**) the possible modification mechanism of PI filaments by O_2_ plasma treatment.

XPS analysis was used to examine the elemental concentration of PI filaments with and without O_2_ plasma treatment. Three high-resolution spectra of C1s, N1s, and O1s regions were collected, and the concentrations of these elements were calculated by peak-area ratios. Table 1 shows the O atom content, and the ratio of O to C (O/C) first increase as the O_2_ plasma treatment time increases from 60 to 180 s and decrease a little afterwards. Conversely, C atom content first decreases and then increase with the increasing plasma treatment time, reaching the bottom peak at 180 s. Moreover, the N atom content has a stable value due to the plasma reactive gas is O_2_. We found that the O_2_ plasma treated PI filaments for 180 s showed the highest O atom content, the highest O/C ratios, and the lowest carbon atom content. Next, Figure 4 shows the C1s core-level XPS spectra of pristine and O_2_ plasma treated PI filaments and the possible plasma working mechanism on the PI filaments’ surface. Table 2 shows the content of functional groups of PI filaments with and without O_2_ plasma treatment. The peaks with bonding energy of 284.5, 285.0, 286.1, and 288.3 eV are assigned to C–C, C–N, C–O, and C=O bonds, respectively [11]. The C–C bond concentrations firstly decrease and then increase with the increasing treatment time. To the contrary, the C–O and C=O bond concentrations increase with the increasing plasma treatment time, and the highest C–O and C=O bond concentrations are obtained with a plasma treatment time of 180 s. However, these functional groups cannot increase continuously if the plasma treatment time is longer than 180 s, mainly due to the degradation of oxygen-containing functional groups caused by excessively long-time plasma treatment. Similarly, with the N atom content, the C–N bond concentration has a stable value as well. FTIR was also applied to elucidate the elemental compositions and polar groups of PI filaments, and the results are in accordance with the XPS conclusion, see Figure S2 in the Supplementary Materials. The radicals formed on PI filaments’ surface by the highly energetic species in the plasma, react with the supplied O_2_, resulting in the secondary formation of the characteristic surface functional groups such as –OH, –COOH, etc. (Figure 4f) [22]. Due to the O_2_ plasma generated polar functional groups, the increased polarity of treated PI filaments can improve the adhesion between PI filaments and the matrix. Based on the above results, it can be concluded that 180 s is the suitable treatment time with respect to the increase of the functional groups of PI filaments.

### 3.3. Contact Angle Analysis

**Figure 5:**
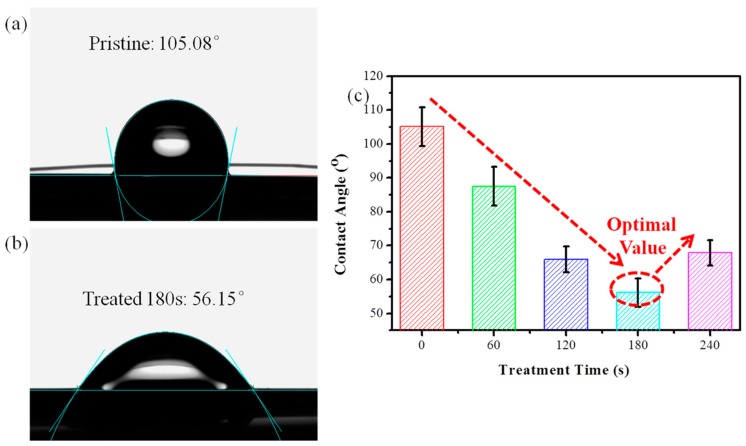
Photographs of water droplets on PI filaments: (**a**) pristine, (**b**) O_2_ plasma treated time of 180 s and (**c**) contact angles for pristine and plasma treated PI filaments at different times.

The contact angle is an important characterization index to evaluate the wettability between the PI filaments and the polar water. A smaller contact angle means a better interfacial adhesion between the PI filaments and the polar matrix. Figure 5 shows the average water contact angles of the pristine, and the O_2_ plasma treated PI filaments. The pristine PI filaments’ surface is hydrophobic with a large static contact angle of 105.08° (Figure 5a). After O_2_ plasma treatment, the PI filaments can obtain a significantly smaller water contact angle, which reaches a bottom peak of 56.15° at the treatment time of 180 s (Figure 5b,c). However, after plasma treatment for 240 s, the water contact angle increased to 67.88° due to the excessively long-time plasma treatment deconstructing the polar groups of the PI filaments. Due to the lotus effect [35], the increased roughness of plasma treated PI fiber tends to increase the water contact angle. Thus, the introduction of polar groups induced by O_2_ plasma is mainly responsible for the improved wettability of the plasma treated PI filaments. The wettability and interfacial adhesion between PI filaments and matrix can be greatly enhanced by O_2_ plasma treatment with the most optimal treatment time of 180 s.

### 3.4. Mechanical Properties

#### 3.4.1. Tensile Properties of PI Filaments

**Figure 6:**
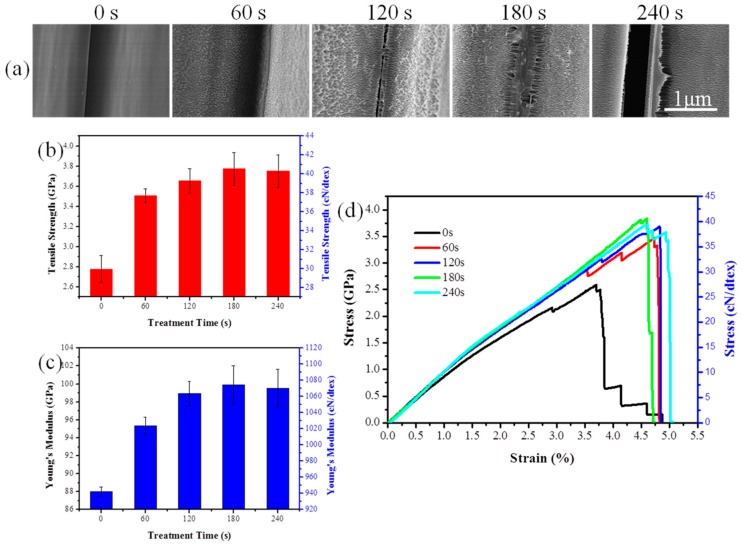
(**a**) Morphologies of the contact area between single PI filament, (**b**) tensile strength, (**c**) tensile modulus and (**d**) stress–strain curves of pristine and plasma treated PI filaments.

Figure 6a shows the morphologies of the contact area between single PI filaments. It can be observed that the contact area between pristine single filaments is well-defined. After being treated for 60 s, there is little bonding between adjacent PI filaments. Then clear bonding traces can be found on the filament surface treated for 120 s. As the treatment time increases to 180 s, the adjacent single filaments are almost bonded together. However, excessive treatment of 240 s will destroy the filament and make a greater gap between the filaments. The mechanical properties of pristine and treated PI filaments are shown in Figure 6b–d. As expected, the tensile strength of PI filaments with a O_2_ plasma treatment time of 180s increase from 2.78 to 3.77 GPa (approximately 36% increase over pristine PI filaments), and the modulus increases from 88 to 100 GPa (approximately 14% increase over pristine PI filaments), suggesting that the O_2_ plasma treatment can enhance the mechanical properties of the PI filaments. That is because the O_2_ plasma treated filaments possess high load transfer efficiency from one filament to another due to the enhancement of bonding properties and mechanical interlocking between the PI filaments. However, a decreasing trend of tensile strength and modulus is observed when the O_2_ plasma treatment time is 240 s, which may be attributed to weak mechanical interlocking caused by the over-etching characteristic on the surface of PI filaments.

#### 3.4.2. Tensile Properties of PI/PAA Composites

**Figure 7:**
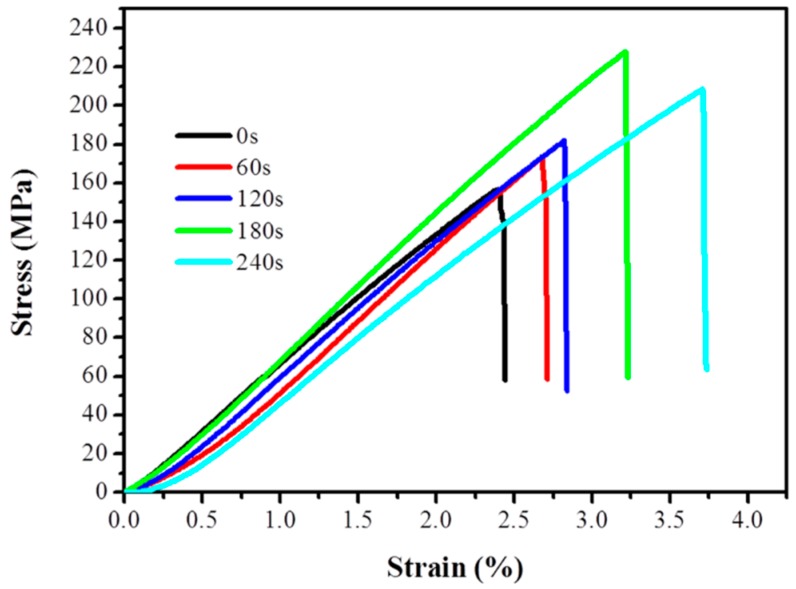
Stress–strain curves of pristine and plasma treated polyimide/poly(amic acid) (PI/PAA) composites.

The tensile properties of PI/PAA composites fabricated using pristine, and O_2_ plasma treated PI filaments are shown in Figure 7. The tensile strength of all O_2_ plasma treated PI/PAA composites improves compared with pristine PI/PAA composite. When the plasma treatment time is 180 s, the tensile strength improvement can be as high as 71.7%. The modification is mostly due to the physically rough and chemically functional groups grafted on the surface of PI filaments, which lead to better adhesion between PI filaments and the matrix.

**Figure 8:**
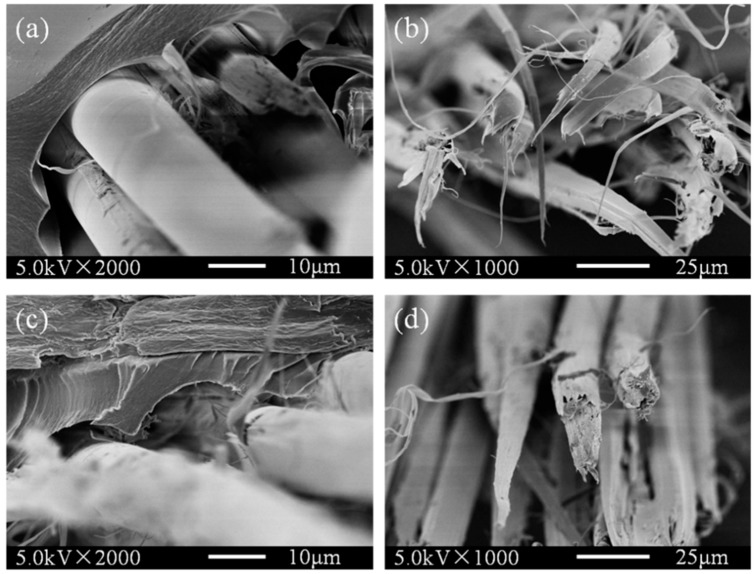
Tensile fracture images of the pristine (**a**,**b**) and plasma treated for 180 s (**c**,**d**) PI filaments self-reinforced composites.

To further study the effects of O_2_ plasma treated on PI filaments, the tensile fracture images of the pristine and 180 s plasma treated PI/PAA composites were obtained as shown in Figure 8. The interface between the pristine PI filaments and PAA matrix is weak and has been separated during the tensile loading, see Figure 8a. The fracture morphology of PI filaments in the pristine PI/PAA composite is split, tabular, and relatively clean with little adhered resin, suggesting the poor interactions between pristine PI filaments and PAA matrix (Figure 8b). As shown in Figure 8c,d, after stretching, the O_2_ plasma treated PI filaments and matrix still stick together, and the fracture morphology changes drastically. The failure mode of O_2_ plasma treated PI/PAA composite is dominated by cohesive failure rather than adhesive failure. The interface between O_2_ plasma treated PI filaments and matrix has a tight bonding, and the filament fracture length is shorter with a lot of resin adhered to the surface, which indicates that the O_2_ plasma treated PI/PAA composites have better interfacial interactions.

## 4. Conclusions

High-performance PI filaments were treated with O_2_ plasma with different time intervals for adhesion enhancement. With the optimal treatment time of 180 s, extensive etching spots appear, and more polar groups are grafted onto the surface of the PI filaments, which implies a greater increase of the wettability. Moreover, the mechanical properties of O_2_ plasma treated PI filaments are improved due to the greater cohesion between the PI single filaments. The adhesion properties between O_2_ plasma treated PI filaments, and PAA matrix is enhanced significantly. The tensile fracture mode of pristine and O_2_ plasma treated PI/PAA composites has been changed from the adhesive failure to cohesive failure. The O_2_ plasma treatment is a promising method for surface modification of high-performance PI filaments as advanced composite reinforcements with better mechanical properties.

## Figures and Tables

**Table 1:** Surface elemental compositions of PI filaments with and without O_2_ plasma treatment.

Treatment Time (s)	Element Content (%)			Atomic Ratio
	C	N	O	O/C
0	84.19	3.33	12.48	0.148
60	80.82	3.43	15.75	0.195
120	77.57	3.49	18.94	0.244
180	75.68	3.39	20.93	0.277
240	77.79	3.42	18.79	0.242

**Table 2:** The content of functional groups of PI filaments with and without O_2_ plasma treatment.

Treatment Time (s)	Concentrations of Correlative Functional Groups (%)			
	C–C (284.5 eV)	C–N (285.0 eV)	C–O (286.1 eV)	C=O (288.3 eV)
0	60.5	24.2	11.2	4.1
60	55.0	24.5	14.8	5.7
120	49.7	24.3	20.2	5.8
180	42.1	24.9	26.4	6.6
240	49.8	24.4	19.4	6.4

## Supplementary Materials

Supplementary materials are available online at http://www.mdpi.com/2073-4360/10/7/695/s1.
