# Supplementary material for: High-Performance Polyimide Filaments and Composites Improved by O2 Plasma Treatment
Source: Polymers (Basel). 2018 Jun 22;10(7):695. doi: 10.3390/polym10070695 (PMC6403617; doi:10.3390/polym10070695)
Supplement: Supplementary file 1 [file polymers-10-00695-s001.pdf]

Table S1. The Comparison Information of Properties of Different Surface Modifications

**Method**	**Increasing Rate (%)**					
	**Roughness**	**Atomic Ratio (O/C)**	**Contact angle**	**Tensile strength of fibers**	**Tensile strength of composites**	**Reference**
O2 Plasma Treatment	+105.34	+87.16	+87.14	+35.61	+71.7	This Article
Cryo-Treatment	+48.76	-	-	-18.37	+30.21	[1]
Nanoparticle Treatment	-	-	+32.72	-	+55.04	[2]
Alkali Treatment	+28.72	-	+22.87	-5	+4.1	[3]

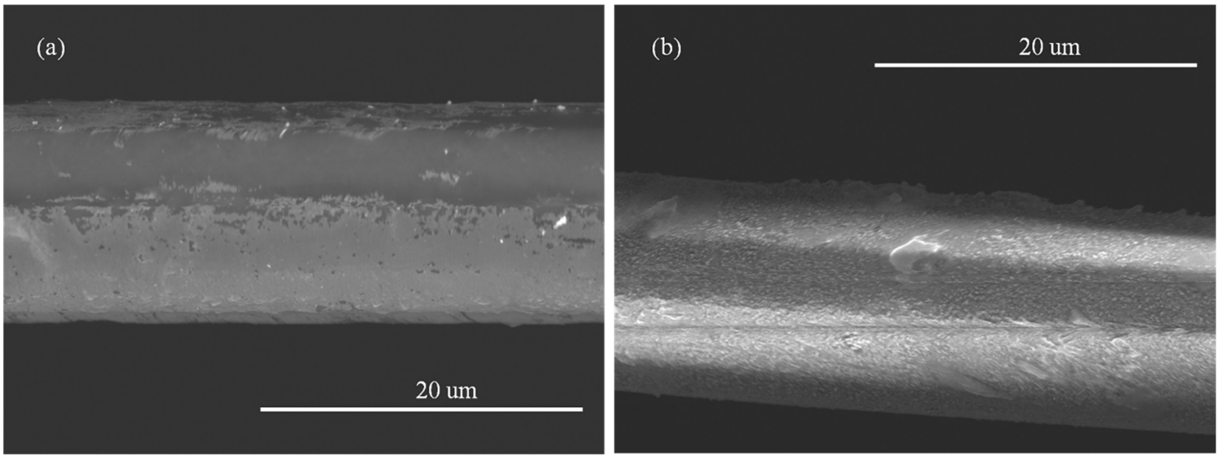

Figure S1. Surface morphologies of PI filaments with plasma treatment of (**a**)120s and (**b**)180s.

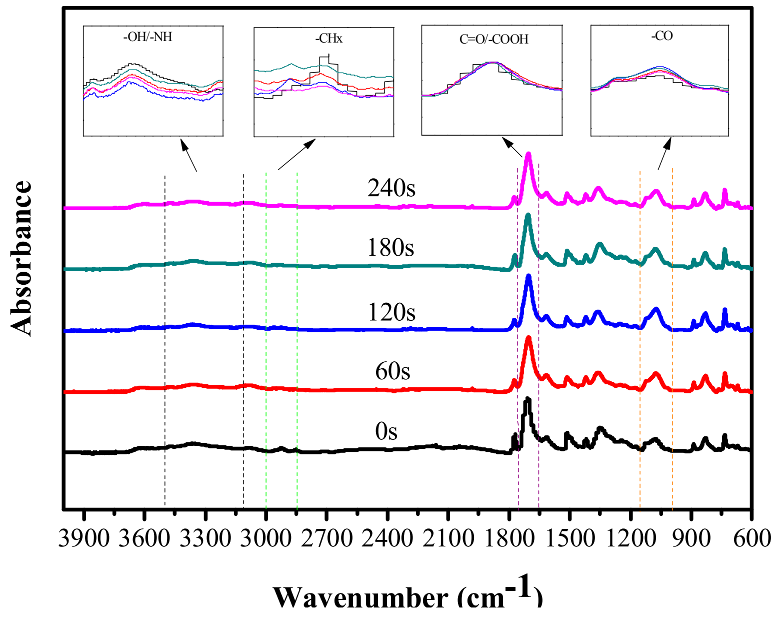

Figure S2. FTIR spectra of pristine and plasma treated PI filaments.

Fourier transform infrared spectroscopy (FTIR) was performed on the Nicolet Nexus 670 using an ATR accessory with a resolution of 2 cm^{-1}. Spectra were obtained with an average of 32 scans, see Figure S2. Comparing with the pristine PI filaments, the oxygen plasma treatment introduces new functional polar groups on the filament surface, including hydroxyl (-OH) and amidogen (-NH) in the range from 3100 to 3500 cm^{-1}, carbon-hydrogen groups (-CHx, such as the -CH2, -CH3) stretch in the range from 2850 to 3000 cm^{-1}, carbonyl (C=O) and carboxyl (-COOH) in the range from 1600 to 1900 cm^{-1} and carbon-oxygen groups (C-O) in the range from 1000 to 1150 cm^{-1} wave number. The transmission peaks around the wave number for these functional polar groups become broader as the increasing treatment time, suggesting the existence of increased functional polar groups. The plasma contains positive and negative ions, electron, among others, that have strong energy to break chemical bonds at the surface and creates radicals, which then produce various oxygen-containing functional polar groups by reacting with oxygen. With the increased functional groups on the surface of the PI filaments, the interfacial adhesion between filament and matrix will be enhanced and the mechanical properties of the obtained composites will be improved accordingly.
